# Postoperative Recovery Trajectories of C-Reactive Protein, Neutrophil-to-Lymphocyte Ratio, and CRP-to-Albumin Ratio After Open Irrigation and Debridement for Native Knee Septic Arthritis: A Descriptive Single-Center Cohort Study

**DOI:** 10.3390/jcm15218311

**Published:** 2026-10-25

**Authors:** Jaemin Lee, YoungHwan Jang, Philsun Park, Jaehyuk Lee, Doo Sup Kim

**Affiliations:** 1 Department of Orthopedic Surgery, Wonju Severance Christian Hospital, Yonsei University Wonju College of Medicine, 20 Ilsan-ro, Wonju 26426, Republic of Korea; megatal12@yonsei.ac.kr (J.L.); jyh3330@naver.com (Y.J.); 2 Department of Orthopedic Surgery, Severance Hospital, Yonsei University College of Medicine, 50-1 Yonsei-ro, Seodaemun-gu, Seoul 03722, Republic of Korea; pkphsun@naver.com; 3 Department of Orthopedic Surgery, Veterans Health Service Medical Center, 53 Jinhwangdo-ro 61-gil, Gangdong-gu, Seoul 05368, Republic of Korea; rhtmrhkd426@gmail.com

**Keywords:** septic arthritis, native knee, irrigation and debridement, C-reactive protein, neutrophil-to-lymphocyte ratio, synovial fluid, reoperation

## Abstract

**Background/Objectives:** The postoperative course of the serum markers that surgeons follow after open irrigation and debridement (I&D) for native knee septic arthritis is poorly defined. We described recovery trajectories of C-reactive protein (CRP), neutrophil-to-lymphocyte ratio (NLR), and CRP-to-albumin ratio (CAR). **Methods:** We retrospectively studied 98 adults treated by open I&D at one center between January 2018 and April 2026. Mixed-effects models with natural splines gave population-average geometric-mean curves for postoperative days 0–42. An exploratory group-by-time interaction compared repeat I&D patients after censoring at the first repeat operation. **Results:** Median age was 77.5 years; median synovial white-cell count was 54,691 cells/µL and median neutrophil percentage 93%; and cultures were positive in 39.8% of patients. Twelve patients (12.2%) underwent repeat I&D, eight during the index admission and four after readmission, and three (3.1%) underwent salvage surgery (minimum estimates). Fitted CRP fell below 5 mg/dL by day 8 and 3 mg/dL by day 13, and CAR fell below 1.0 by day 12; fitted NLR reached 3 by day 22, but its confidence band did not exclude non-attainment within 42 days. Milestones were 1 to 13 days later in culture-confirmed subgroups. In exploratory, underpowered analyses censored at reoperation, neither preoperative levels nor decline slopes clearly distinguished repeat I&D patients (CRP interaction *p* = 0.78); an uncensored model’s nominal CRP association largely disappeared. **Conclusions:** CRP, NLR, and CAR showed distinct population-average postoperative trajectories after open I&D. These descriptive curves may assist expectation-setting and study design but should not be used as stand-alone indicators of infection control or joint outcome. Multicenter prospective studies with standardized sampling and functional endpoints are needed.

## 1. Introduction

Native knee septic arthritis is a destructive joint infection [1] whose mortality remains high in older adults [2,3]. Delay worsens cartilage loss, function, and survival [4], so guidelines call for urgent surgical drainage with targeted antimicrobial therapy [5]. Whether arthroscopic or open drainage is preferable remains debated [6,7,8,9]; open irrigation and debridement (I&D) is widely used for advanced disease. Swedish national registry data show knee debridement for septic arthritis rising, mostly in patients aged 65 to 84 years [10], and a contemporaneous cohort concluded that arthrotomy remains necessary for complex or advanced disease [11].

After debridement, surgeons judge recovery with serum inflammatory markers. Early postoperative C-reactive protein (CRP) has been linked to treatment failure [12]; the neutrophil-to-lymphocyte ratio (NLR) has diagnostic and prognostic value in native joint infection [13]; and the CRP-to-albumin ratio (CAR), which adds nutritional status, has been informative in periprosthetic infection [14,15]. In native knee septic arthritis, however, postoperative behavior has been described mainly for CRP at early or fixed time points, with conflicting results [12,16,17]; a Korean series risk-stratified failure after arthroscopic debridement [18], and the long-term course was mapped only recently [19]. Multi-week trajectories after open I&D, modeled from irregular real-world sampling, are lacking.

We therefore describe the clinical and synovial-fluid profile of a single-center open-I&D cohort, model CRP, NLR, and CAR recovery with mixed-effects models, examine factors associated with length of stay, and explore whether trajectories differ by repeat I&D. To our knowledge, multi-week NLR and CAR trajectories after open I&D of the native knee have not been described. A methodological observation shows how uncensored post-reoperation measurements can produce an apparent early-marker association. The study is descriptive; it does not develop or validate a prognostic tool.

## 2. Materials and Methods

### 2.1. Study Design and Participants

**Figure 1:**
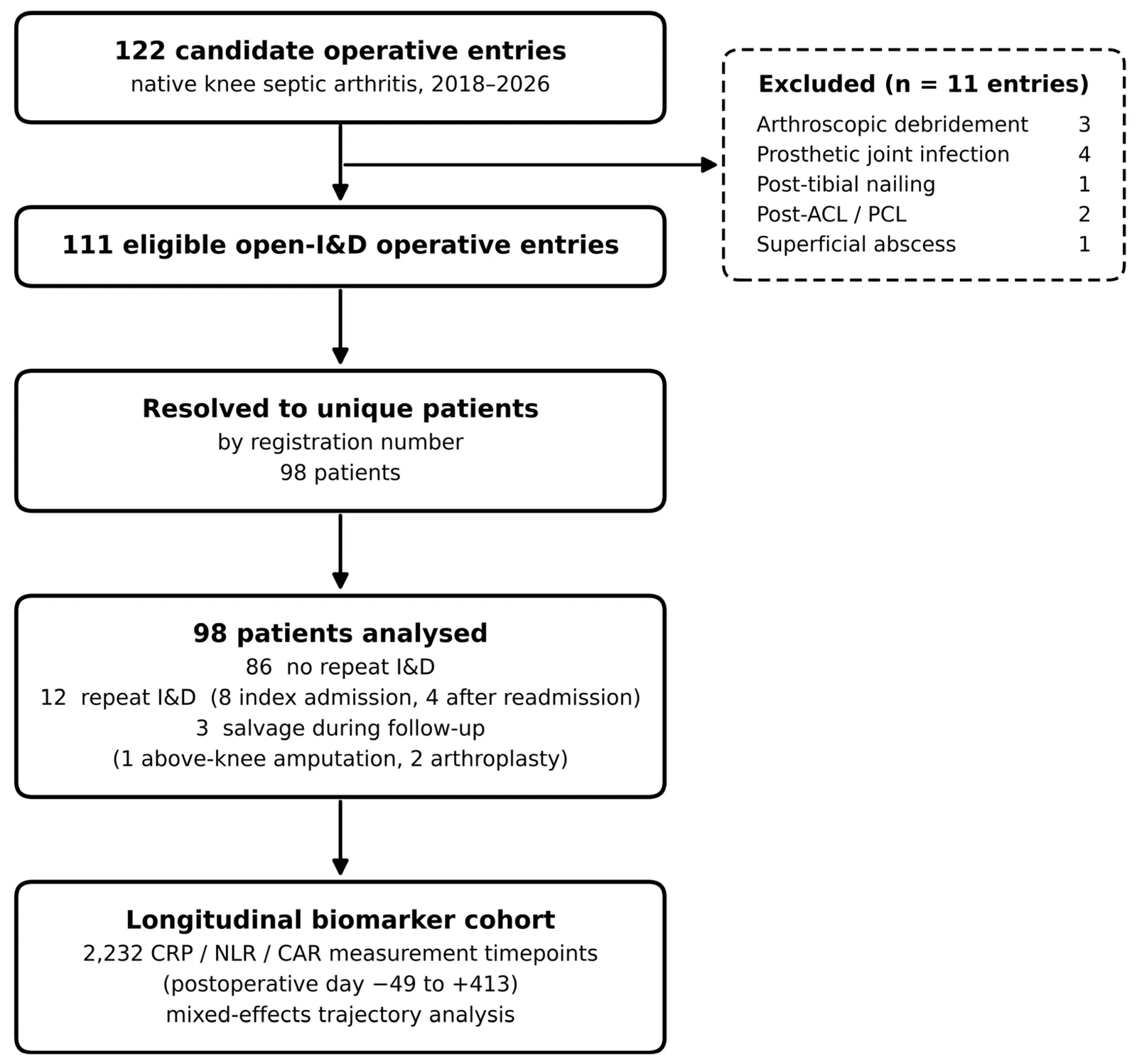
Patient flow. Upper-box counts are operative entries: 111 from 98 patients; the 12 with more than one are the repeat I&D patients, and only each patient’s earliest open I&D was analyzed. ACL, anterior cruciate ligament; I&D, irrigation and debridement; PCL, posterior cruciate ligament.

This retrospective single-center cohort study follows the Strengthening the Reporting of Observational Studies in Epidemiology statement. We reviewed consecutive adults aged 18 years or older who underwent open I&D for native knee septic arthritis with index operations from 21 January 2018 to 24 April 2026. The diagnosis was clinical, based on presentation, synovial fluid analysis, culture, and intraoperative findings, without requiring a positive culture or a fixed synovial white-cell threshold, consistent with recognized culture-negative native septic arthritis [20]. Culture-negative joints were diagnosed chiefly from the synovial white-cell count of the diagnostic aspirate and the gross appearance of the aspirate and intraoperative specimens (Supplementary Table S5). Joints with crystals were included on the same clinical grounds as other joints; no separate criterion was applied (their findings are given in Section 3.2). Histopathology was not abstracted. Open I&D was the institutional default throughout the study period, without separate indications; patients previously operated on elsewhere for the same episode were included (Section 3.1). We excluded arthroscopic debridement alone, periprosthetic joint infection, infection after a separate index procedure (tibial nailing or cruciate ligament reconstruction), and superficial abscess. The earliest open I&D per patient was the index operation and time origin; 111 eligible operative entries came from 98 patients (Figure 1).

### 2.2. Surgical Management and Data Sources

All index procedures used a 300 mmHg tourniquet, a midline incision, and medial parapatellar arthrotomy; after synovectomy, the joint was lavaged with 12 to 15 L of saline with dilute povidone-iodine and hydrogen peroxide, and a drain was placed. Motion and weight-bearing began early as tolerated. Every patient, including those without culture growth, received antimicrobial therapy under infectious-disease guidance, culture-directed when a pathogen was isolated and empirical otherwise, with the end of treatment decided jointly with that service. Antibiotic exposure before sampling and the duration of intravenous therapy could not be determined reliably, partly because some patients may have been treated elsewhere before referral. Aspirate, intraoperative joint fluid, and tissue were cultured; tissue culture was routine, but the number and type of specimens were not standardized. Methicillin resistance in *Staphylococcus aureus* was defined by oxacillin or cefoxitin susceptibility. Any growth in a joint specimen was counted as culture-positive, including skin commensals (coagulase-negative staphylococci, *Corynebacterium*, *Bacillus*), recorded without adjudication; any other organism was termed a recognized pathogen. Blood cultures did not define culture positivity; one patient with growth in blood culture only was classed as culture-negative. Demographic, comorbidity, operative, and laboratory data came from the electronic medical record, and prior intra-articular injection, acupuncture or bloodletting, and symptom duration from the admission history, as was any surgery for the presenting episode at another hospital. An exposure not mentioned in an available admission history was recorded as not documented, not as absent; such exposures were reported descriptively and were neither imputed nor modeled. Symptom duration was recorded as one week or less, longer than one week, long-standing without a stated episode duration, or not documented. Laboratory data were retrieved from up to 49 days before the index operation to the last available measurement (latest value dated 26 May 2026, on postoperative day 413). This collection window is distinct from the analytical window: the trajectory models used postoperative days 0 to 42 only (1200 of 2010 postoperative laboratory records). Measurements after day 42 were used only to describe laboratory follow-up, individual first-observed crossings occurring after day 42 (Section 3.3), and discharge values for admissions extending beyond day 42.

### 2.3. Variables and Definitions

The primary variables were the postoperative trajectories of CRP (mg/dL; 1 mg/dL = 10 mg/L), NLR (absolute neutrophil count divided by lymphocyte count), and CAR (CRP in mg/dL divided by same-day albumin in g/dL). The prognostic nutritional index (PNI) was 10 × albumin (g/dL) + 0.005 × lymphocyte count (/µL) from the preoperative draw closest to surgery. Measurements were indexed to postoperative day. Synovial white-cell count, neutrophil percentage, and crystals under compensated polarized light were recorded from the diagnostic aspiration and tabulated against the customary cut-offs of more than 50,000 and more than 25,000 cells/µL and more than 90% neutrophils for comparability only [21]. Repeat I&D and salvage surgery were ascertained from operative records and the institutional operative schedule. Repeat I&D was a further irrigation and debridement of the index knee; manipulation under anesthesia for stiffness was not counted, as identified from the recorded procedure and its documented indication (Supplementary Table S6). No second-look surgery was scheduled; repeat I&D was performed when serial CRP rose again after its initial decline. Each repeat I&D was classified by timing (index admission or after readmission) and by the indication documented in the operative and progress notes: a renewed CRP rise without other documented signs, or clinical signs of persistent or recurrent infection (effusion, wound drainage, fever, or pain). Salvage surgery was defined as conversion to arthroplasty, arthrodesis, or amputation, recorded with its interval and indication. No composite endpoint was constructed. Length of stay covered the index admission, and the last in-admission CRP was the final value on or before discharge. Laboratory follow-up ran to the last available measurement. Repeat I&D and salvage surgery were ascertained from the institutional operative schedule through 30 April 2026, so each patient’s operative ascertainment ran from the index operation to that date (median 38.1 months, IQR 27.6–69.1; shorter than 12 months in 15 patients and shorter than 3 months in 3). Outpatient clinical follow-up was not abstracted.

### 2.4. Statistical Analysis

Continuous variables are reported as median with interquartile range (IQR) and categorical variables as counts with percentages, with Wilson 95% confidence intervals for key proportions. Spearman correlation was used to relate preoperative serum markers to synovial indices, and culture-positive and recognized-pathogen joints were compared with the remainder by Mann–Whitney U and Fisher exact tests. Denominators reflect available data without imputation; no sample-size calculation was made.

Trajectories over days 0 to 42 were modeled with linear mixed-effects models [22]: each log-transformed marker was regressed on a natural cubic spline of postoperative day [23] with a patient random intercept, fitted by restricted maximum likelihood (full specification in the Supplementary Methods). A natural spline was chosen because the decline is non-linear and of unknown shape; four degrees of freedom (knots at days 0, 14, 28, and 42) were fixed a priori, with three and five as sensitivity analyses. Testing times were assumed ignorable given postoperative day and the random effect (missing at random; see Limitations). The submitted analysis included a redundant intercept, which left the coefficients unidentified and caused the reported convergence warning; without it, all models converged with a positive-definite fixed-effect covariance, and the fitted curves changed by at most 0.4%, with identical milestone days. A random slope for postoperative day improved fit for all three markers (likelihood-ratio test, 2 df, all *p* < 0.001) and left the primary milestones unchanged for CRP and CAR and two days later for NLR, but after about day 21 its CAR curve kept declining (0.27 against 0.55 at day 42), bringing the non-primary CAR 0.5 crossing to day 23, and late prediction intervals widened (Supplementary Figure S1 and Table S2). The random-intercept model was not selected on fit; it is retained as the reporting anchor for continuity with the figures and milestones of the version under review, and the better-fitting random-slope model is shown alongside it (curves in Supplementary Figure S1; milestones and random-effects covariance in Table S2). The two specifications gave the same early CRP and CAR milestones (CRP 5 and 3 mg/dL, CAR 1.0) and NLR crossings of 3 differed by two days, and interpretation rests on these early milestones; late CAR values and late prediction intervals differed and are treated as model-dependent. Back-transformed fits are geometric means, shown with model-free window medians. Confidence intervals were the 2.5th–97.5th percentiles of 1000 patient-level cluster-bootstrap resamples [24], each retained only if its fit converged (all did); prediction intervals added between-patient and residual variance (Supplementary Methods).

Sensitivity analyses used one averaged measurement per patient per window; excluded repeat I&D patients or those whose repeat operation followed clinical signs of infection; censored at the first repeat operation; varied the spline degrees of freedom; and, because the diagnosis was clinical, restricted the analysis to culture-positive and recognized-pathogen subgroups or excluded crystal-positive joints (Supplementary Table S3). Time to a reference value was the day the fitted geometric mean crossed it, with an interval from the crossing days of the lower and upper confidence limits (not reached if the upper limit was not crossed by day 42); these conventional laboratory anchors are not derived cut-offs or definitions of normalization. Each patient’s own crossing was the first observed value below the reference, reported with the preceding sampling interval and separated into crossings by day 42 and later, with an interval-midpoint Kaplan–Meier median as a sensitivity estimate and a variant censored at the first repeat operation. Fitted population crossings and individual first-observed crossings are different quantities and are reported side by side.

Factors associated with length of stay were examined in an exploratory multiple linear regression on log-transformed stay; because four repeat operations followed discharge and could not lengthen the index stay, a sensitivity model coded only repeat I&D during the index admission. In an exploratory analysis, a group-by-time interaction on a linear postoperative-day term with a patient random slope compared patients with and without repeat I&D, censoring all measurements from the first repeat operation onward because reoperation re-elevates markers. The three marker interactions were tested against a Bonferroni threshold of 0.017 and, with only 12 events, treated as hypothesis-generating; no prediction model was built. Baseline comparisons by repeat I&D are unadjusted and descriptive. Two-sided *p* < 0.05 denoted nominal significance. Analyses were performed in Python 3.11.9 with statsmodels 0.14.6, patsy 1.0.2, SciPy 1.17.1, NumPy 2.4.4, and pandas 3.0.2; the analysis code is available from the corresponding author on reasonable request.

## 3. Results

### 3.1. Cohort Characteristics

Ninety-eight patients were included (Figure 1; Table 1); most were elderly, comorbid, admitted through the emergency department, and of American Society of Anesthesiologists (ASA) class III or higher. Symptom duration was documented in 46 patients, 30 of whom (65.2%) presented within one week. A prior intra-articular injection was documented in 21 (21.4%), a minimum estimate. Surgery at another hospital for the presenting episode was documented in seven patients (7.1%; arthroscopic surgery (recorded as I&D, debridement, or synovectomy) in six and open debridement in one), also a minimum estimate from the 82 available admission notes; excluding them moved the fitted milestones by at most one day (Supplementary Table S3). Cultures were positive in 39 patients (39.8%), with a recognized pathogen in 28 (28.6%), most often *S. aureus*. Preoperative inflammatory markers were markedly elevated (Table 2).

### 3.2. Synovial Fluid Analysis

Aspiration was attempted in all 98 patients and yielded fluid in 95 (96.9%); with three dry taps and five clotted samples, a white-cell count was available in 90 and a differential in 91, showing a neutrophil-predominant leukocytosis (Table 2). Crystals were found in nine joints, three of them culture-positive (two with a recognized pathogen). In the six culture-negative crystal-positive joints, the synovial white-cell count was 52,660 to 90,200 cells/µL in five (neutrophils 80% to 94%) and 29,190 cells/µL in one (87%), in which the intraoperative findings supported infection; none underwent repeat I&D. Preoperative serum CRP, NLR, and CAR did not correlate with the synovial white-cell count or neutrophil percentage (Table 2). Synovial counts did not differ between culture-positive and culture-negative joints (median 57,586 against 52,850 cells/µL, *p* = 0.72; neutrophils 92% against 93%, *p* = 0.83) or by recognized pathogen, whereas preoperative CRP, NLR, and CAR were higher in culture-positive patients (Supplementary Table S5).

### 3.3. Postoperative Marker Trajectories

**Figure 2:**
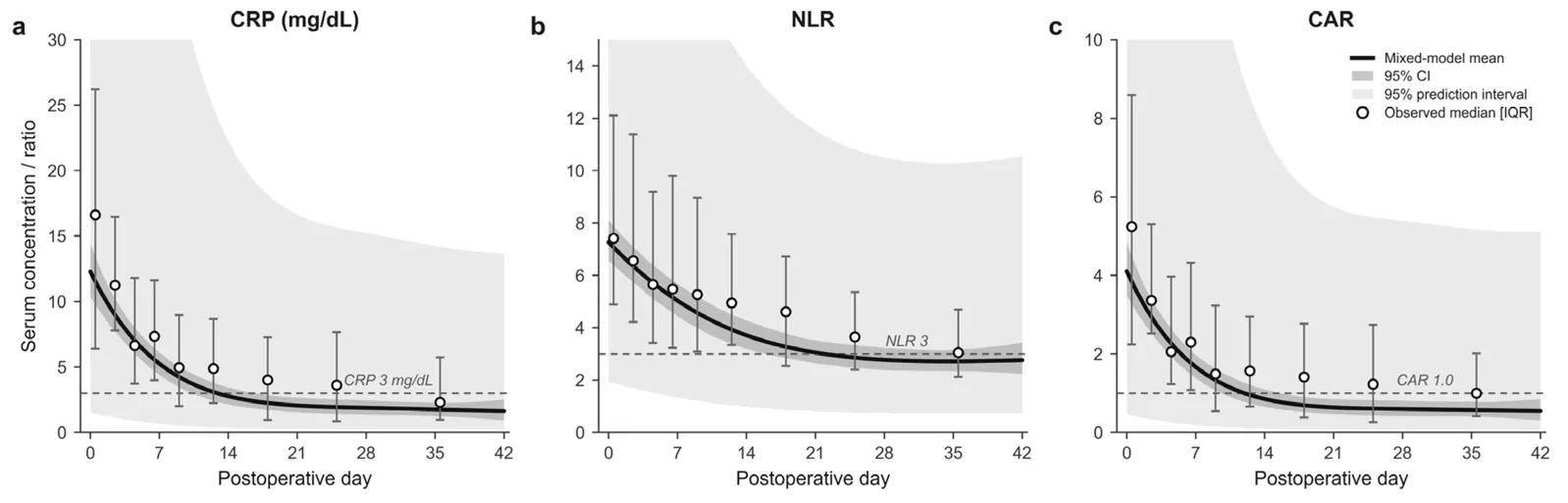
Recovery trajectories of (**a**) CRP, (**b**) NLR, and (**c**) CAR over days 0–42. Line, fitted geometric mean (random-intercept model); dark band, 95% confidence interval (cluster bootstrap); light band, 95% prediction interval; circles, observed window median (IQR); dashed line, reference value (CRP 3 mg/dL; NLR 3; CAR 1.0). Y-axes are truncated: the upper prediction bound exceeds the plotted range until day 10 for CRP and CAR and day 12 for NLR. Random-slope curves: Supplementary Figure S1. CAR, CRP-to-albumin ratio; CRP, C-reactive protein; IQR, interquartile range; NLR, neutrophil-to-lymphocyte ratio.

All three markers declined after surgery (Figure 2), with CRP in the first two postoperative days exceeding its preoperative median (Table 2). The fitted curves fell steeply and then flattened; window medians ran higher later, reaching each reference value one to several weeks later. Fitted milestones are given in Table 2 (landmark values in Supplementary Table S4); NLR reached 3 last and least precisely: its upper confidence limit stayed above 3 throughout, so attainment of 3 within 42 days was not demonstrated. Milestones moved by at most three days across sampling and modeling specifications and by at most one day after excluding crystal-positive joints, but later in the culture-positive (n = 39) and recognized-pathogen (n = 28) subgroups: by one to three days for CRP, three for CAR, and 5 to 13 for NLR, with the culture-positive NLR crossing falling at day 35 (Supplementary Table S3). Between-patient variability was substantial: at day 14 the fitted CRP was 2.8 mg/dL (95% CI 2.1–3.4), with a 95% prediction interval of 0.3 to 22.2 mg/dL.

The fitted curve and the observed patients agreed within one to two days at the earliest-reached reference values and diverged at slower-resolving ones (Table 2, Panel D). For NLR below 3, the fitted crossing was day 22, whereas 71 patients were first observed below 3 by day 42. Fitted CRP never reached 1 mg/dL by day 42; 47 of 97 patients were observed below it by day 42 and a further 24 only later.

### 3.4. Clinical Outcomes

Twelve patients (12.2%) underwent repeat I&D: eight during the index admission (median postoperative day 13, range 4–22; one had a second in-admission repeat) and four after readmission (median day 48.5, range 41–65). Five followed a renewed CRP rise without other documented signs, all during the index admission, and seven followed clinical signs of persistent or recurrent infection, three during the index admission and all four after readmission (Table 3; per-operation listing, including the two manipulations under anesthesia that were not counted, in Supplementary Table S6). Three patients (3.1%) underwent salvage surgery 15 to 46 months after the index operation: one above-knee amputation, for persistent infection, and two conversions to total knee arthroplasty, for post-infectious joint destruction, a recognized later course [25,26,27,28] (Table 3). Both rates are minimum estimates, as only procedures at our institution were captured. Laboratory follow-up spanned a median of 111 days. In the exploratory length-of-stay model, stay was longer with higher ASA class (35% per class) and with repeat I&D, partly mechanically, but was not associated with age, sex, diabetes, or preoperative CRP (*p* = 0.06) (Table 3). Coding only the eight patients reoperated during the index admission as repeat I&D strengthened the repeat I&D association (ratio 2.11, 95% CI 1.34 to 3.32, against 1.81 for all 12) without materially changing the other estimates; the four patients reoperated only after readmission also had longer index stays than those without repeat I&D (median 22 against 15 days), so the association may partly reflect severity. Preoperative PNI was available in 96 patients (median 38.9, IQR 35.2 to 44.3), 95 of whom had complete covariate data; in these 95, it raised the model R^2^ from 0.223 to 0.275, each point being associated with a 2.4% shorter stay (ratio 0.976, 95% CI 0.958 to 0.995, *p* = 0.015; Table 3, Panel C); the association is descriptive, not predictive. At discharge (last in-admission CRP; a median of 13 days after surgery), only 23 of 97 patients were below 1 mg/dL (Table 2, Panel E), and 81 had further CRP measurements afterward, so falling below the lower reference values was largely an outpatient event.

### 3.5. Exploratory Trajectory–Outcome Analysis

These analyses were exploratory and underpowered. Preoperative CRP, NLR, CAR, albumin, and erythrocyte sedimentation rate did not differ between the repeat I&D groups, nor did culture positivity, recognized pathogens, or *S. aureus*, although a recognized pathogen was grown in half of the repeat I&D patients against about a quarter of the others (*p* = 0.096) (Table 4). After censoring at the first repeat operation, decline slopes did not differ (interaction *p* = 0.78 for CRP, 0.064 for NLR, and 0.52 for CAR), with bootstrap confidence intervals spanning zero; with 12 events, meaningful differences were neither demonstrated nor excluded. A naive uncensored random-intercept model gave a nominal CRP difference (β = 0.142 per 10 days, *p* = 0.04); a random slope widened its standard error without reducing the estimate (β = 0.179, *p* = 0.15), and censoring more than halved it (β = 0.049, 95% CI −0.402 to 0.414, *p* = 0.78; Supplementary Figure S2). The seven repeat operations that followed clinical signs of infection were too few for inference (Supplementary Table S3).

## 4. Discussion

CRP, NLR, and CAR followed distinct declining trajectories after open I&D for native knee septic arthritis. Window medians ran above the fitted curves later; cross-sectional medians and model-based geometric means weight patients differently, and the reason for the gap was not tested. In exploratory analyses, neither preoperative levels nor decline slopes clearly distinguished patients who required repeat I&D, and with 12 events, this is not evidence that the markers lack prognostic value.

**Post-event measurements and apparent prognostic associations.** That the effect size of the naive CRP association, not merely its precision, fell only when post-reoperation values were censored (Section 3.5) suggests post-reoperation re-elevation as a likely source, though with 12 events, the censored estimate remains compatible with the uncensored one and the cause is not established. In our practice, reoperation was itself triggered by a renewed CRP rise, so CRP before the event is partly its trigger and CRP after it partly its consequence; when post-event values enter an uncensored trajectory model, slower apparent decline in the operated group can follow from the operation itself. Studies associating early CRP with failure used values at fixed early days or within 24 h [12,16], which mostly precede any repeat operation, so this mechanism is unlikely to explain their findings. Prediction studies using serial markers should state how post-event measurements were handled and should prefer landmark designs.

This exploratory null finding is consistent with a cohort of 97 native septic knees managed by open I&D, in which the CRP trend over the first two postoperative days was not associated with repeat washout and only younger age and *S. aureus* on aspiration culture were associated with failure [17]. In our cohort, neither age (median 77.0 against 78.0 years) nor *S. aureus* differed between the groups (Table 4). A two-day trend and multi-week slopes are not equivalent, and repeat washout occurred in 43% there against 12.2% here, so the two null findings are concordant rather than confirmatory.

Previous work has largely examined markers at isolated time points [12,13,18,19]. Our contribution is descriptive: continuous population-average curves from irregularly sampled routine markers, including the multi-week course of NLR and CAR. CAR has been studied in septic arthritis only as single landmark values, at two weeks in the native shoulder [29] and at admission in children [30]. The most comparable knee study associated a raised initial white-cell count and a CRP decline below 20% at 24 h with secondary surgery [16]; it used a single early predictor, whereas we asked whether multi-week slopes separate outcomes once re-elevation is removed.

Elective arthroplasty differs from septic arthritis in disease context and surgical indication, so its postoperative marker course is not a directly comparable reference population; it illustrates only that knee surgery itself contributes to the early postoperative marker response. After total knee arthroplasty, mean NLR fell from 5 at day 2 to 3.5 at day 4 and 2.6 at day 21 in 587 patients [31], with a similar course after hip arthroplasty [32]. Fitted NLR here ran higher but converged (6.53, 5.87, and 3.05 at the same days), whereas fitted CRP was lower early (95.5 against 163 mg/L at day 2) and about twice as high late (20.6 against 9 mg/L at day 21; stated in mg/L to match the comparison series), plausibly reflecting the smaller surgical insult of arthrotomy early and the infection itself late; CRP and the erythrocyte sedimentation rate stay elevated for weeks even after uncomplicated arthroplasty [33]. The comparison is qualitative: our geometric means run systematically below arithmetic means for right-skewed markers, which is conservative for the late CRP difference and exaggerates the early one, and populations, operations, and eras differ.

This is also one of the few Asian open-I&D native-knee series to characterize the diagnostic synovial fluid: it showed the typical neutrophil-predominant leukocytosis, with crystals coexisting in a minority, so identifying crystals does not exclude infection. That serum markers did not track synovial indices is consistent with their capturing different facets of disease, the systemic inflammatory response and the local intra-articular burden [13]. Our median stay exceeded that of a contemporaneous open-arthrotomy cohort (16 versus 11.5 days), consistent with an older case mix [11]. Culture-negative joints had synovial counts similar to those of culture-positive ones but lower systemic markers, consistent with infection that was less severe systemically or partly treated before sampling [20], although similar synovial counts do not exclude diagnostic misclassification. Cohort composition did influence the estimated tempo: milestones came later in culture-confirmed subgroups, most for NLR, whose fitted mean crossed 3 at day 35 rather than day 22 (point estimates; even in the whole cohort, the confidence band did not exclude non-attainment within 42 days), whereas excluding crystal-positive joints changed them by at most one day. The whole-cohort curve therefore describes this clinically diagnosed cohort; it should not be used as a reference for microbiologically confirmed infection, in which recovery may be slower, and the culture-negative subgroup should be interpreted cautiously.

**Clinical implications.** The implications concern postoperative surveillance, not surgical technique, and are specific to open I&D, since CRP recovers differently after arthroscopic continuous irrigation [9]. First, CRP may rise above its preoperative value in the first days, so an early rise is not in itself a departure from the usual course. Second, CRP gave the most interpretable trajectory, falling below 5 mg/dL by about the second week. Third, NLR and CAR, available from routine blood tests, were described alongside CRP, but their incremental value for clinical decisions was not evaluated; NLR in particular was imprecise, its band still including 3 at day 42, and it is affected by corticosteroids, physiological stress, non-infectious illness, and age [32]. Fourth, departure of an individual patient’s values from the fitted mean curve is not validated evidence of treatment failure or an indication for repeat surgery, and no marker threshold for such decisions is proposed. The curves describe the expected tempo of recovery for counseling and supply effect sizes and variance components for powering landmark studies (Supplementary Tables S2 and S4); they describe the cohort, not an individual patient (Table 2, Panel D), and are not validated individual thresholds. They describe systemic marker recovery, not recovery of the joint: a declining marker does not establish eradication of infection, and markers may settle despite persisting local pathology or a poor functional result. They must be read alongside serial examination, wound status, pain, range of motion, microbiology, imaging where appropriate, and the surgeon’s assessment. The reference values are conventional anchors, not definitions of normalization.

Several limitations apply. The study is retrospective and single-center, and the timing of repeat debridement, antibiotics, and discharge was not protocolized. With 12 repeat I&D events, seven following clinical signs of infection, every outcome analysis is underpowered and hypothesis-generating. Because repeat I&D was conducted when serial CRP rose again, the endpoint is partly defined by the marker under study: postoperative CRP cannot be evaluated here as an independent predictor of repeat I&D, the any-repeat endpoint is not equivalent to treatment failure, and the clinical-sign subgroup was too small for inference. Censoring shortened the exposed group’s trajectory, so the censored interaction is under-informed rather than evidence of parallel decline. A composite failure endpoint could not be constructed because readmission, culture-confirmed recurrence, and mortality were not uniformly ascertainable, and no functional, range-of-motion, pain, cartilage, or radiographic outcome was available. Severity was available only as preoperative markers, synovial counts, ASA class, and comorbidity; no stage, such as the Gächter classification [34], was recorded. The diagnosis was clinical; 60.2% of joints were culture-negative, and histopathology was not abstracted; similar synovial counts across culture groups do not exclude misclassification, and non-infectious inflammatory arthritis cannot be excluded in every case. Microbiology, prior injection, and symptom duration were not modeled as determinants of marker kinetics, which 12 events cannot support, though each is clinically relevant [12,18,34,35,36,37]; symptom duration was documented in fewer than half of patients, and prior injection is a minimum estimate because explicit denial was not systematically recorded, although the interval from onset to surgery is associated with reoperation [38]. Antibiotic choice and adequacy, a principal determinant of how quickly inflammation settles [39], were not assessed, and neither exposure before sampling nor treatment duration could be determined reliably, so the curves describe recovery under routine infectious-disease-guided care; unlike a contemporaneous cohort [11], we could not report an empiric antibiogram or in-hospital mortality. The length-of-stay model is exploratory and descriptive, since repeat I&D during the index admission mechanically lengthens that admission. Late CAR values and prediction intervals depended on the random-effects specification: with a random slope, CAR fell below 0.5 by day 23, and late prediction intervals widened, so late-window estimates, unlike the early milestones, are model-dependent. Surgery elsewhere before referral was documented in seven patients but may be under-recorded; outpatient clinical follow-up was not abstracted, and operative ascertainment was shorter than 12 months in 15 patients operated late in the study period. Laboratory testing was not protocolized and was plausibly more frequent in complicated courses. The mixed model accommodates unequal sampling but cannot fully correct for selective testing: if testing depended on the unobserved value (missing not at random), the fitted curve could be biased, and the direction of any such bias cannot be established from these data; the one-measurement-per-window analysis addresses frequency, not this selection. Finally, the curves come from one elderly, comorbid Korean tertiary population, limiting generalizability, although this is the group in which native septic arthritis is most lethal [2,3]; milestone days are conditional on this case mix, and the window medians (Table 2) are a model-free complement to the fitted curves.

Future work should validate these trajectories in multicenter prospective cohorts with standardized sampling (an illustrative, not validated, schedule would be postoperative days 1–2 for the early CRP rise, 3–5 and 7 for the steep decline, 14 near the fitted CRP and CAR milestones, and 28 and 42 for the late phase, where estimates were least precise), integrate microbiological, antimicrobial, functional, and nutritional data, and use landmark designs that avoid reverse causation.

## 5. Conclusions

In this single-center retrospective cohort, CRP, NLR, and CAR showed distinct population-average postoperative trajectories after open irrigation and debridement for native knee septic arthritis. These descriptive curves may assist postoperative expectation-setting and the design of future studies, but they should not be used as stand-alone indicators of infection control or joint outcome. In the exploratory, underpowered analyses, no marker clearly distinguished repeat I&D patients, an endpoint partly triggered by renewed CRP rise and not equivalent to treatment failure, and an apparent early CRP signal in an uncensored model was largely removed by censoring at reoperation. Multicenter prospective studies with standardized sampling, complete outcome ascertainment, and functional endpoints are needed before clinical adoption.

## Figures and Tables

**Table 1:** Baseline characteristics (*n* = 98).

Characteristic	Value
Age (years), median (IQR)	77.5 (66.0–84.0)
Male sex, n (%)	57 (58.2)
Height (cm), median (IQR)	162.0 (153.8–168.9)
Weight (kg), median (IQR)	60.0 (54.0–70.0)
Body mass index (kg/m^2^), median (IQR)	23.8 (21.6–26.1)
Admission via emergency department, *n* (%)	73 (74.5)
Symptom duration, *n* (%)	
1 week or less (documented)	30 (30.6)
Longer than 1 week (documented)	16 (16.3)
Long-standing symptoms, episode duration not stated	5 (5.1)
Not documented	47 (48.0)
Prior intra-articular knee injection, *n* (%)	
Documented	21 (21.4)
Not documented in an available admission history	61 (62.2)
No admission history available	16 (16.3)
Prior acupuncture or bloodletting (any site), *n* (%)	
Documented	5 (5.1)
Not documented in an available admission history	77 (78.6)
No admission history available	16 (16.3)
ASA class, median (IQR)	3.0 (2.0–3.0)
ASA I/II/III/IV, n	3/27/57/11
Hypertension, n (%)	64 (65.3)
Diabetes mellitus, n (%)	35 (35.7)
Cardiovascular disease, n (%)	22 (22.4)
Chronic kidney disease, n (%)	13 (13.3)
Cerebrovascular disease, n (%)	9 (9.2)
Peripheral arterial disease, n (%)	2 (2.0)
At least one comorbidity, n (%)	76 (77.6)
Current smoker, n (%)	15 (15.3)
Former smoker, n (%)	7 (7.1)
Never smoker, n (%)	76 (77.6)
Affected side—right/left, n (%)	50 (51.0)/43 (43.9)
Anesthesia—general/spinal, n (%)	66 (67.3)/32 (32.7)
Length of stay (days), median (IQR)	16.0 (11.2–27.0)
Microbiology	
Culture-positive (aspirate, joint fluid, or tissue), n (%)	39 (39.8)
Recognized pathogen (excluding probable contaminants), n (%)	28 (28.6)
*Staphylococcus aureus*, n	14
MSSA/MRSA, n	11/3
Gram-negative bacilli, n	10
β-hemolytic *Streptococcus*, n	2
*Enterococcus* species, n	2
Fungal (*Aspergillus*, yeast), n	2
Coagulase-negative staphylococci, n	9
*Corynebacterium* or *Bacillus*, n	3
Polymicrobial (two or more organisms), n	6

Data are median (IQR) or n (%). Side indeterminate in 5. Symptom duration documented in 46 (median 4.5 days [IQR 2.0–14.0]). Denial of injection, acupuncture, or bloodletting was not recorded systematically, so documented exposures are minimum estimates. Culture-negative: 58 no growth, one blood isolate only. Recognized pathogens exclude skin commensals; categories overlap. ASA, American Society of Anesthesiologists physical status; IQR, interquartile range; MRSA/MSSA, methicillin-resistant/-susceptible *Staphylococcus aureus*.

**Table 2:** Preoperative serum and synovial markers and postoperative recovery. (Panel **A**): Preoperative serum markers (closest value on or before surgery). (Panel **B**): Preoperative synovial fluid (diagnostic aspiration). (Panel **C**): Observed postoperative median (IQR) by window (fitted curves in Figure 2). (Panel **D**): Fitted curve versus individual patients. (Panel **E**): Markers at discharge from the index admission.

(**A**)											
**Marker**				***n***				**Median** **(IQR)**			
CRP (mg/dL)				96				11.4 (6.3–18.7)			
NLR				98				7.2 (4.4–11.7)			
CAR				94				3.4 (1.8–6.6)			
Albumin (g/dL)				96				3.3 (2.9–3.8)			
WBC (×10^3^/µL)				98				10.1 (7.5–12.9)			
ESR (mm/hr)				92				52.5 (34.8–72.5)			
(**B**)											
**Measure**				**Value**				**95% CI**			
Aspiration yielding fluid, n/N (%)				95/98 (96.9)				91.4–99.0			
White-cell count (cells/µL), median (IQR) (n = 90)				54,691 (35,476–88,577)				—			
Neutrophils (%),median (IQR) (n = 91)				93 (88–95)				—			
WBC > 50,000/µL, n/N (%)				51/90 (56.7)				46.4–66.4			
WBC > 25,000/µL, n/N (%)				79/90 (87.8)				79.4–93.0			
Neutrophils > 90%, n/N (%)				57/91 (62.6)				52.4–71.9			
Crystals identified, n				9				—			
Aspiration was attempted in all 98 patients; three dry taps and five clotted samples left white blood cell counts and differentials in 90 and 91 aspirates, respectively. Crystals: monosodium urate 7, calcium pyrophosphate 1, and basic calcium phosphate 1. Serum markers did not correlate with synovial count or neutrophil percentage (Spearman |ρ| ≤ 0.16, all *p* ≥ 0.13).											
(**C**)											
**Postoperative Day**		**CRP (mg/dL)**				**NLR**				**CAR**	
0–1		16.6 (6.4–26.2)				7.4 (4.9–12.1)				5.2 (2.2–8.6)	
2–3		11.2 (7.8–16.5)				6.6 (4.2–11.4)				3.4 (2.5–5.3)	
4–5		6.7 (3.7–11.8)				5.7 (3.4–9.2)				2.1 (1.2–4.0)	
6–7		7.3 (4.0–11.6)				5.5 (3.2–9.8)				2.3 (1.1–4.3)	
8–10		5.0 (2.0–9.0)				5.3 (3.1–9.0)				1.5 (0.5–3.2)	
11–14		4.9 (2.2–8.7)				5.0 (3.3–7.6)				1.6 (0.7–3.0)	
15–21		4.0 (0.9–7.3)				4.6 (2.5–6.7)				1.4 (0.4–2.8)	
22–28		3.6 (0.9–7.7)				3.6 (2.4–5.4)				1.2 (0.3–2.7)	
29–42		2.3 (1.0–5.7)				3.0 (2.1–4.7)				1.0 (0.4–2.0)	
Measurements and patients per window: Supplementary Table S1. Fitted-curve milestones (95% band; concordant in the one-per-window analysis; Supplementary Table S4). CRP < 5 mg/dL day 8 (6–10), <3 mg/dL day 13 (11–17), <1 mg/dL not reached; CAR <1.0 day 12 (10–16), <0.5 not reached; NLR <3 day 22, imprecise: the band is compatible with 3 from day 16 but never excludes non-attainment; <2 not reached. CAR 1.0 = 10 with CRP in mg/L.											
(**D**)											
**Reference Value**	**Fitted Curve Crosses**		**Patients Observed Below, n/N**		**By Day 42/Later, n**		**Individual Median Day** **(IQR)**		**Kaplan–Meier Median**		**Sampling Interval, Median (Max) Days**
CRP < 5 mg/dL	day 8		89/97		82/7		7 (5–14)		6		3 (44)
CRP < 3 mg/dL	day 13		83/97		72/11		11 (6–24)		10		3 (44)
CRP < 1 mg/dL	not reached by day 42		71/97		47/24		22 (14–52)		31		8 (77)
NLR < 3	day 22		83/98		71/12		11 (5–26)		13.5		3 (91)
NLR < 2	not reached by day 42		64/98		52/12		18 (10–37)		28		4 (43)
CAR < 1.0	day 12		78/97		67/11		11 (6–27)		11.5		3 (82)
CAR < 0.5	not reached by day 42		65/97		51/14		14 (8–40)		26.5		4 (91)
Columns 2 and 5 are different quantities: the fitted crossing belongs to the population-average curve; the individual day is the first value observed below the reference, bounded by testing times (sampling interval = median and maximum gap between the last value at or above and the first below). Reference values are conventional anchors, not definitions of normalization. Patients who were never observed below remain in the denominator (CRP 8, 14, 26; NLR 15, 34; CAR 19, 32). Kaplan–Meier medians (interval midpoint; censored at the last value) differ from first-observed medians. Fitted day-42 values: CRP 1.63 mg/dL, NLR 2.76, CAR 0.55. Censoring at the first repeat operation moved individual medians by at most one day.											
(**E**)											
**Measure**						**Value**					
Postoperative day of the last in-admission CRP, median (IQR)						13 (8–21)					
Last in-admission CRP (mg/dL), median (IQR)						2.76 (1.13–5.18)					
Last in-admission CRP < 5 mg/dL, n/N (%)						71/97 (73.2)					
Last in-admission CRP < 3 mg/dL, n/N (%)						53/97 (54.6)					
Last in-admission CRP < 1 mg/dL, n/N (%)						23/97 (23.7)					
Patients with any CRP measured after discharge, n						81					
Length of stay: Table 1. Discharge was not protocolized. Patients measured after discharge had a median of four CRP values; one had no in-admission postoperative CRP. CRP in mg/dL (1 mg/dL = 10 mg/L); CAR computed with CRP in mg/dL. CAR, CRP-to-albumin ratio; CRP, C-reactive protein; ESR, erythrocyte sedimentation rate; IQR, interquartile range; NLR, neutrophil-to-lymphocyte ratio; WBC, white blood cell count.											

**Table 3:** Clinical outcomes and factors associated with length of stay. (Panel **A**): outcomes (n = 98). (Panel **B**): exploratory linear regression of log length of stay (n = 96; two lacked preoperative CRP). (Panel **C**): model with the preoperative prognostic nutritional index added (n = 95).

(**A**)		
**Outcomes**	***n*** **(%)**	**Wilson 95% CI**
No repeat irrigation and debridement	86 (87.8)	79.8–92.9
Any repeat irrigation and debridement	12 (12.2)	7.1–20.2
By timing: during the index admission (postoperative day 13 [4–22])	8 (8.2)	4.2–15.3
By timing: after discharge and readmission (postoperative day 48.5 [41–65])	4 (4.1)	1.6–10.0
By indication: renewed CRP rise without other documented signs	5 (5.1)	2.2–11.4
By indication: clinical signs of persistent or recurrent infection	7 (7.1)	3.5–14.0
Salvage surgery	3 (3.1)	1.0–8.6
Above-knee amputation (15 months; persistent infection)	1 (1.0)	—
Conversion to total knee arthroplasty (16 and 46 months; post-infectious joint destruction)	2 (2.0)	—
From the institutional operative schedule; minimum estimates. No second look was scheduled; repeat I&D followed a renewed CRP rise. Indication = first repeat operation per patient (operative and progress notes); clinical signs = effusion, wound drainage, fever, or pain. Manipulations under anesthesia are not counted (per-operation listing: Supplementary Table S6). One patient had a second in-admission repeat. Postoperative day: median (range); months from the index operation. Laboratory follow-up median 111 days (IQR 45–254); operative outcomes through 30 April 2026 (operative ascertainment median 38.1 months [IQR 27.6–69.1]; under 12 months in 15 patients).		
(**B**)		
**Predictor**	**Ratio (95% CI)**	***p***
Age (per year)	1.002 (0.992–1.012)	0.71
Male sex	0.976 (0.765–1.245)	0.84
Diabetes mellitus	0.872 (0.674–1.128)	0.29
ASA class (per class)	1.347 (1.125–1.614)	0.002
Repeat I&D	1.814 (1.244–2.644)	0.002
Preoperative CRP (per mg/dL)	1.012 (0.999–1.026)	0.063
Ratio = exp(β); above 1, longer stay. The repeat I&D association is partly mechanical: a repeat operation during the index admission lengthens that admission by definition. R^2^ 0.223 (refit on the 95 rows of Panel C) and 0.275 (Panel C). Repeat I&D here includes all 12 patients, four of whom were reoperated only after readmission. Sensitivity analysis coding only the eight patients reoperated during the index admission as repeat I&D: ratio 2.109 (1.339–3.320), *p* = 0.002, with ASA 1.321 (1.103–1.582) and preoperative CRP 1.010 (0.997–1.023, *p* = 0.12), R^2^ 0.229; with PNI added (*n* = 95): repeat I&D 1.844 (1.170–2.908) and PNI 0.977 (0.958–0.995), R^2^ 0.279. ASA, American Society of Anesthesiologists physical status; CRP, C-reactive protein; I&D, irrigation and debridement.		
(**C**)		
**Predictor**	**Ratio (95% CI)**	***p***
Age (per year)	0.998 (0.989–1.008)	0.75
Male sex	0.992 (0.780–1.262)	0.95
Diabetes mellitus	0.894 (0.694–1.152)	0.38
ASA class (per class)	1.261 (1.048–1.518)	0.015
Repeat I&D	1.626 (1.115–2.371)	0.012
Preoperative CRP (per mg/dL)	1.008 (0.995–1.021)	0.23
Preoperative PNI (per point)	0.976 (0.958–0.995)	0.015
PNI = 10 × albumin (g/dL) + 0.005 × lymphocyte count (/µL); available in 96, of whom 95 had complete covariate data and were analyzed. PNI tracked stay more closely than albumin or lymphocytes alone (Spearman ρ −0.43, −0.36, −0.28) and did not differ by repeat I&D (38.5 versus 39.1, *p* = 0.58). Descriptive, not causal or predictive. PNI, prognostic nutritional index.		

**Table 4:** Exploratory trajectory–outcome analysis: comparison by repeat irrigation and debridement. (Panel **A**): Characteristics, preoperative markers, and microbiology. (Panel **B**): Postoperative decline slope (group-by-time interaction, random slope, censored at the first repeat operation).

(**A**)				
**Variable**	**Repeat I&D (n = 12)**	**No repeat I&D (n = 86)**		***p***
Age (years), median	77.0	78.0		0.32
ASA class, median	3.0	3.0		0.55
Preoperative CRP (mg/dL), median	12.2	10.8		0.79
Preoperative NLR, median	11.7	6.8		0.18
Preoperative CAR, median	4.5	3.4		0.56
Preoperative albumin (g/dL), median	3.2	3.4		0.53
Preoperative ESR (mm/hr), median	54.0	52.5		0.88
Male sex, n/N	8/12	49/86		0.76
Diabetes mellitus, n/N	7/12	28/86		0.11
Chronic kidney disease, n/N	2/12	11/86		0.66
Hypertension, n/N	7/12	57/86		0.75
Culture-positive, n/N	7/12	32/86		0.21
Recognized pathogen, n/N	6/12	22/86		0.096
*Staphylococcus aureus*, n/N	2/12	12/86		0.68
(**B**)				
**Marker**	**Interaction β per 10 Days (95% CI)**		***p***	
CRP	0.049 (−0.402 to 0.414)		0.78	
NLR	−0.168 (−0.405 to 0.016)		0.064	
CAR	0.150 (−0.298 to 0.589)		0.52	
Mann–Whitney U or Fisher exact tests; exploratory, none significant. Panel A *p*-values are unadjusted and descriptive; Panel B used a Bonferroni threshold of 0.017. With 12 events, neither panel can demonstrate or exclude clinically important differences. Preoperative n (repeat/no repeat): CRP 11/85, NLR 12/86, CAR 10/84, albumin 12/84, and ESR 10/82; Hodges–Lehmann shifts all spanned zero. CIs are from the cluster bootstrap. After censoring, all 12 repeat patients contributed (58, 98, and 56 measurements for CRP, NLR, and CAR). ASA, American Society of Anesthesiologists physical status; CAR, CRP-to-albumin ratio; CRP, C-reactive protein; ESR, erythrocyte sedimentation rate; I&D, irrigation and debridement; NLR, neutrophil-to-lymphocyte ratio.				

## Data Availability

The datasets generated and analyzed during the current study are available from the corresponding author on reasonable request. The data are not publicly available owing to patient-privacy restrictions. The analysis code is likewise available from the corresponding author on reasonable request.

## Supplementary Materials

The following supporting information can be downloaded at: https://www.mdpi.com/article/10.3390/jcm15218311/s1, Supplementary Methods: Mixed-effects trajectory model; Table S1: Measurements and contributing patients per postoperative-day window; Table S2: Mixed-effects model diagnostics and random-effects sensitivity; Table S3: Robustness of the fitted recovery milestones to cohort definition, censoring, spline flexibility, and diagnostic certainty; Table S4: Fitted geometric-mean marker values at landmark postoperative days, with 95% confidence and prediction intervals; Table S5: Synovial fluid and preoperative serum markers by microbiological status; Table S6: Reoperations evaluated for repeat I&D classification; Figure S1: Random-intercept versus random-slope trajectories; Figure S2: Effect of censoring at the first repeat operation.

## References

[1] Elsissy J.G., Liu J.N., Wilton P.J., Nwachuku I., Gowd A.K., Amin N.H. (2020). Bacterial septic arthritis of the adult native knee joint: A review. JBJS Rev..

[2] Choi H.J., Yoon H.K., Oh H.C., Hong J.H., Choi T., Park S.H. (2022). Mortality of septic knee arthritis in Korea: Risk factors analysis of a large national database. Sci. Rep..

[3] Taubert L., Neijhoft J., Wölfl C.G. (2026). Primary septic arthritis in the elderly—A forgotten killer? Five-year survival and risk factors in a retrospective cohort study. Eur. J. Trauma Emerg. Surg..

[4] Mathews C.J., Weston V.C., Jones A., Field M., Coakley G. (2010). Bacterial septic arthritis in adults. Lancet.

[5] Ravn C., Neyt J., Benito N., Abreu M.A., Achermann Y., Bozhkova S., Coorevits L., Ferrari M.C., Gammelsrud K.W., Gerlach U.J. (2023). Guideline for management of septic arthritis in native joints (SANJO). J. Bone Jt. Infect..

[6] Kennedy G.E.M., Tharmaseelan A., Phillips J.R.A., Evans J.T., Kunutsor S.K. (2024). Does arthroscopic or open washout in native knee septic arthritis result in superior post-operative function? A systematic review and meta-analysis of randomised controlled trials and observational studies. Syst. Rev..

[7] Nudelman B.M., Piple A.S., Ferkel R.D. (2025). Arthroscopy is at least as effective as arthrotomy for treatment of septic arthritis in adults: A systematic review of large and intermediate joints. Am. J. Sports Med..

[8] Puzzitiello R.N., Agarwalla A., Masood R., Bragg J., Hanna J., Pagani N.R., Salzler M.J. (2024). Arthroscopic treatment is a safe and effective alternative to open treatment for acute septic arthritis of the native knee: A systematic review. Arthroscopy.

[9] Xiao J., Wu W., Lin S., Zheng Y., Lin K., You T., Wu Q., Shen F., Yang X., Han X. (2025). A comparison of arthroscopy combined with continuous irrigation, arthroscopic debridement alone, and open arthrotomy for the treatment of septic arthritis of the native knee. BMC Musculoskelet. Disord..

[10] Schmidt V., Magnéli M., Axenhus M. (2026). Trends in surgical management of septic arthritis of the knee: A 16-year observational study of 4,809 surgeries in Sweden. BMC Musculoskelet. Disord..

[11] Argüelles-Alegría A., Tafoya-Arreguín G.A., Herrera-Fuentes L.F., Madrigal-Rodríguez M., Martínez-Ruíz J.J., Pérez Manzo R., De Arcos-Jiménez J.C., Briseno-Ramirez J. (2026). Real-world management of native knee septic arthritis at a university hospital, 2018–2025: A retrospective cohort with comparative surgical outcomes and development of a weighted-incidence syndromic combination antibiogram. J. Orthop..

[12] Straub J., Lingitz M.T., Apprich S., Staats K., Windhager R., Böhler C. (2023). Early postoperative laboratory parameters are predictive of initial treatment failure in acute septic arthritis of the knee and shoulder joint. Sci. Rep..

[13] Varady N.H., Schwab P.E., Kheir M.M., Dilley J.E., Bedair H., Chen A.F. (2022). Synovial fluid and serum neutrophil-to-lymphocyte ratio: Novel biomarkers for the diagnosis and prognosis of native septic arthritis in adults. J. Bone Jt. Surg. Am..

[14] Yigit Ş., Akar M.S., Şahin M.A., Arslan H. (2021). Periprosthetic infection risks and predictive value of C-reactive protein/albumin ratio for total joint arthroplasty. Acta Biomed..

[15] Wu H., Pan L., Meng Z., Liu H., Yang X., Cao Y. (2022). C-reactive protein (CRP)/albumin-to-globulin ratio (AGR) is a valuable test for diagnosing periprosthetic joint infection: A single-center retrospective study. J. Orthop. Traumatol..

[16] Yeo Q.Y., Li A.T.C., Cuttilan A.N., Low J.C.C., Ho S.W.L. (2022). Raised initial total white cell count and lower post-operative decline of C reactive protein increases the risk of secondary surgery in septic arthritis of the native knee. Knee Surg. Sports Traumatol. Arthrosc..

[17] Korrapati A., Zhu K.Y., Kent W.T. (2026). Predictors of repeat washout in the isolated septic knee. J. Knee Surg..

[18] Byun J., Jung M., Chung K., Jung S.H., Jang H., Choi C.H., Kim S.H. (2025). Risk factors for failure to eradicate infection after single arthroscopic debridement in septic arthritis of a native knee joint. Yonsei Med. J..

[19] Clement R.G.E., Wong S.J., Hall A., Howie S.E.M., Simpson A.H.R.W. (2024). The long-term time course of septic arthritis. Bone Jt. Open.

[20] Paz Z., Fowler M.L., Zhu C., Lieber S.B., Moore A., Shmerling R.H. (2020). Patients with surgically treated culture-negative native joint septic arthritis have less severe disease and better outcomes. Infect. Dis..

[21] Margaretten M.E., Kohlwes J., Moore D., Bent S. (2007). Does this adult patient have septic arthritis?. JAMA.

[22] Laird N.M., Ware J.H. (1982). Random-effects models for longitudinal data. Biometrics.

[23] Harrell F.E. (2015). Regression Modeling Strategies: With Applications to Linear Models, Logistic and Ordinal Regression, and Survival Analysis.

[24] Efron B., Tibshirani R.J. (1993). An Introduction to the Bootstrap.

[25] Bettencourt J.W., Wyles C.C., Fruth K.M., Osmon D.R., Hanssen A.D., Berry D.J., Abdel M.P. (2021). Outcomes of primary total knee arthroplasty following septic arthritis of the native knee: A case-control study. J. Bone Jt. Surg. Am..

[26] Kwon Y.U., Lee C.R., Kim C.W., Jung S.H., Baek S.H. (2025). Outcomes of two-stage total knee arthroplasty following septic arthritis of the native knee: A propensity score-matched analysis. Knee.

[27] Sanka S.K., Tahir M., Lakpriya S., De C., Oni T., Pierce T.P. (2025). Total hip or knee arthroplasty in patients with a history of native joint septic arthritis: A meta-analysis and systematic review of risk factors of prosthetic joint infection. Clin. Orthop. Surg..

[28] Russo A., Migliorini F., Giustra F., Bosco F., Massè A., Burastero G. (2024). Two-stage total joint replacement for hip or knee septic arthritis: Post-traumatic etiology and difficult-to-treat infections predict poor outcomes. Arch. Orthop. Trauma Surg..

[29] Seong J.E., Lee J.A., Lee Y.S., Kim J.H., Ku N.S., Choi J.Y., Yeom J.S., Jeong S.J. (2025). Clinical characteristics and predictors of outcomes in septic arthritis of the shoulder: A 16-year study. Bone Jt. J..

[30] Chen X., Liu Y., Zou C., Sun Y., Zhuang T., Zhao C., Sun Q., Li M., Zheng W., Wang X. (2025). Predictive value of the C-reactive protein to albumin ratio in the treatment of septic arthritis in young children: A retrospective study. Orthop. Traumatol. Surg. Res..

[31] Yombi J.C., Schwab P.E., Thienpont E. (2016). Neutrophil-to-lymphocyte ratio (NLR) distribution shows a better kinetic pattern than C-reactive protein distribution for the follow-up of early inflammation after total knee arthroplasty. Knee Surg. Sports Traumatol. Arthrosc..

[32] Mundama M., Van Cauter M., Detrembleur C., Cornu O., Dubuc J.E., Yombi J.C. (2020). Neutrophil-to-lymphocyte ratio (NLR) distribution shows an advantage compared to C-reactive protein (CRP) for the early inflammation monitoring after total hip arthroplasty. Acta Orthop. Belg..

[33] Incesoy M.A., Demirkiran C.B., Kaya H.B., Geckalan M.A., Tak A.Y., Elmali N., Yildiz F., Uzer G. (2025). Natural course of postoperative C-reactive protein and erythrocyte sedimentation rate in unilateral and simultaneous bilateral total knee arthroplasty. BMC Musculoskelet. Disord..

[34] De Franco C., Artiaco S., de Matteo V., Bistolfi A., Balato G., Vallefuoco S., Massè A., Rosa D. (2022). The eradication rate of infection in septic knee arthritis according to the Gächter classification: A systematic review. Orthop. Rev..

[35] Fernandes L.S., Yagi A.J., Santos Netto A., Salles M.J., Oliveira V.M., Cury R.P.L. (2023). *S. aureus* is associated with a greater need for reoperation in septic arthritis of the knee. Acta Ortop. Bras..

[36] Pesante B.D., Salimi M., Miller W.L., Young H.L., Jenkins T.C., Parry J.A. (2024). Associations with unplanned repeat irrigation and debridement of native septic arthritis. Eur. J. Orthop. Surg. Traumatol..

[37] Linden G.S., Lipson S.E., Desai V., Krivicich L.M., Puzzitiello R.N., Gabriel D., Alswang J.M., Wurcel A.G., Ryan S.P., Chen A.F. (2025). Injection drug use is associated with repeat irrigation and debridement in native joint septic arthritis. J. Orthop..

[38] Bozca M.A., Salmanoğlu S.E., Altuntaş Y., İpek E., Hürmeydan Ö.M., Kural C. (2026). The impact of surgical timing on reoperation after arthroscopic versus open treatment of acute native knee septic arthritis. Int. Orthop..

[39] Gjika E., Beaulieu J.Y., Vakalopoulos K., Gauthier M., Bouvet C., Gonzalez A., Morello V., Steiger C., Hirsiger S., Lipsky B.A. (2019). Two weeks versus four weeks of antibiotic therapy after surgical drainage for native joint bacterial arthritis: A prospective, randomised, non-inferiority trial. Ann. Rheum. Dis..

