# Supplementary material for: Postoperative Recovery Trajectories of C-Reactive Protein, Neutrophil-to-Lymphocyte Ratio, and CRP-to-Albumin Ratio After Open Irrigation and Debridement for Native Knee Septic Arthritis: A Descriptive Single-Center Cohort Study
Source: J Clin Med. 2026 Oct 25;15(21):8311. doi: 10.3390/jcm15218311 (PMC13764029; doi:10.3390/jcm15218311)
Supplement: Supplementary file 1 [file jcm-15-08311-s001.zip › Supplementary_Materials_REV2.pdf]

Supplementary material

Postoperative recovery trajectories of C-reactive protein, neutrophil-to-lymphocyte ratio, and CRP-to-albumin ratio after open irrigation and debridement for native knee septic arthritis: a descriptive single-center cohort study

Supplementary Methods. Mixed-effects trajectory model

Specification. Each marker y (CRP in mg/dL, NLR, or CAR) was analyzed on the natural-logarithm scale. For patient i and postoperative day t, the model was

$$\log y(ij) = \mathbf{s}(t(ij)) + u(i) + \varepsilon(ij),$$

where $\mathbf{s}(\cdot)$ is a natural cubic regression spline of postoperative day with four degrees of freedom (knots at postoperative days 0, 14, 28, and 42, identically for all three markers), whose four basis functions sum to one and therefore carry the overall level without a separate intercept, $u(i) \sim N(0, \tau^2)$ is a patient-level random intercept, and $\varepsilon(ij) \sim N(0, \sigma^2)$ is the residual. Models were fitted by restricted maximum likelihood over postoperative days 0–42 (statsmodels MixedLM; patsy basis `cr(POD, df=4) - 1`). Fitted values were exponentiated and therefore represent geometric means.

Convergence (Reviewer 1). The submitted analysis added a separate intercept to a basis whose functions already sum to one, giving a five-column design of rank four: the fixed-effect coefficients were unidentified (hence the non-positive-definite covariance and failed convergence criterion), whereas the fitted curve was identified (hence identical curves across optimizers). Without the redundant intercept, all six models (three markers, two random-effects specifications) converged under limited-memory BFGS with positive-definite fixed-effect covariance (smallest eigenvalue 7.2×10^{-4} to 3.3×10^{-3}); the random-intercept curves differed from those originally reported by at most 0.4%, with identical milestone days. Variance components changed slightly (e.g., τ^2 0.68 rather than 0.73 for CRP; Supplementary Table S2) because the restricted likelihood of the rank-deficient design was misstated.

Intervals. The 95% confidence interval of the fitted geometric mean was the percentile interval of a patient-level (cluster) bootstrap of 1,000 resamples (fixed seed); a resample was retained only if its fit converged, which all did for each marker and specification. Asymptotic (Wald) intervals were similar, the bootstrap intervals being wider near the window edges. The 95% prediction interval was the fitted log-mean ± 1.96 × $\sqrt{(V\_boot + V\_u(t) + \sigma^2)}$, back-transformed, where V_boot is the bootstrap variance of the fitted log-mean and V_u(t) the between-patient variance: τ^2 under the random intercept and $\mathbf{z}(t)^T \mathbf{G}\, \mathbf{z}(t)$, with $\mathbf{z}(t) = (1, t/42)$, under the random slope.

Random-effects specification. A random slope for postoperative day (scaled as day/42, unstructured 2 × 2 covariance **G**) improved fit for all three markers (likelihood-ratio χ^2 118.65, 62.26, and 117.79 for CRP, NLR, and CAR; 2 df, all $p < 0.001$). Under both specifications (Supplementary Figure S1 and Table S2) the curves were nearly superimposable to about day 21, the confidence bands overlapped at every day, and the primary milestones were identical for CRP and CAR and two days later for NLR (day 24). After day 21, however, the random-slope CAR curve kept declining (0.27 against 0.55 at day 42), placing the non-primary CAR 0.5 crossing at day 23 rather than beyond day 42, and its prediction intervals widen because between-patient variance grows with time. The random-intercept model was not selected on fit; it is retained as the reporting anchor for continuity with the figures and milestones of the version under review, and the better-fitting random-slope model is shown alongside it (curves in Supplementary Figure S1; milestones and random-effects covariance in Table S2). Interpretation rests on the early milestones, on which the two specifications agree

(identical for CRP and CAR, two days apart for NLR); late CAR values and late prediction intervals depend on the specification and are shown graphically under both.

Observation process. Observation times are treated as ignorable: given postoperative day and the random effect, testing is assumed unrelated to the unobserved marker value (missing at random). Testing was not protocolized, so this may not hold (see Limitations).

Supplementary Table S1. Measurements and contributing patients per postoperative-day window

Postoperative day	CRP measurements (patients)	NLR measurements (patients)	CAR measurements (patients)
0–1	60 (47)	184 (98)	59 (46)
2–3	79 (72)	111 (85)	77 (70)
4–5	87 (80)	114 (86)	84 (77)
6–7	57 (52)	75 (59)	56 (51)
8–10	86 (72)	115 (76)	80 (68)
11–14	108 (71)	141 (75)	102 (67)
15–21	113 (67)	163 (69)	96 (54)
22–28	74 (45)	107 (46)	60 (38)
29–42	99 (58)	130 (61)	74 (39)
**Total measurements**	**763**	**1,140**	**688**
**Unique patients**	**97**	**98**	**97**

Measurements per window (distinct patients in parentheses); patients recur across windows. CAR, CRP-to-albumin ratio; CRP, C-reactive protein; NLR, neutrophil-to-lymphocyte ratio.

Supplementary Table S2. Mixed-effects model diagnostics and random-effects sensitivity

Marker	Measurements	Patients	Residual variance (σ^2)	Random-intercept variance (τ^2)	ICC	Milestone days (95% band), random intercept	Milestone days (95% band), random slope
CRP	763	97	0.43	0.68	0.61	<5 day 8 (6–10); <3 day 13 (11–17); <1 not reached	<5 day 8 (7–10); <3 day 13 (11–16); <1 not reached
NLR	1,140	98	0.17	0.28	0.63	<3 day 22 (16–not reached); <2 not reached	<3 day 24 (16–not reached); <2 not reached
CAR	688	97	0.42	0.82	0.66	<1.0 day 12 (10–16); <0.5 not reached	<1.0 day 12 (10–15); <0.5 day 23 (17–not reached)

Variances on the natural-log scale (random-intercept model); ICC = $\tau^2/(\tau^2 + \sigma^2)$. Milestone day = first day the fitted geometric-mean curve crossed the reference; band = crossing days of the lower and upper 95%

confidence limits; "not reached" = no crossing by day 42. Random-slope comparison: Supplementary Methods. Random-slope **G** (intercept variance, covariance, slope variance; slope on day/42): CRP 0.345, 0.348, 2.008; NLR 0.281, −0.008, 0.443; CAR 0.421, 0.556, 3.555. CAR, CRP-to-albumin ratio; CRP, C-reactive protein; ICC, intraclass correlation coefficient; NLR, neutrophil-to-lymphocyte ratio.

Supplementary Table S3. Robustness of the fitted recovery milestones to cohort definition, censoring, spline flexibility, and diagnostic certainty

Panel A. Measurement-aggregation, subgroup, and censoring sensitivity (natural cubic spline, 4 degrees of freedom).

Analysis	Patients (n)	CRP <5 / <3 mg/dL	NLR <3	CAR <1.0
Primary (all patients, as reported)	97–98	day 8 / day 13	day 22	day 12
One averaged measurement per patient per window	97–98	day 8 / day 13	day 22	day 12
Exclude all repeat-I&D patients	85–86	day 8 / day 13	day 22	day 12
Exclude patients whose first repeat operation followed clinical signs of persistent or recurrent infection	90–91	day 8 / day 13	day 23	day 12
Censor measurements on/after the first repeat operation	97–98	day 8 / day 13	day 22	day 12

Panel B. Spline flexibility sensitivity (primary cohort).

Spline degrees of freedom	CRP <5 / <3 mg/dL	NLR <3	CAR <1.0
3	day 8 / day 14	day 22	day 13
4 (primary)	day 8 / day 13	day 22	day 12
5	day 8 / day 13	day 25	day 12

Panel C. Diagnostic-certainty sensitivity (natural cubic spline, 4 degrees of freedom unless noted).

Analysis	Patients (n)	CRP <5 / <3 mg/dL	NLR <3	CAR <1.0
Primary (all patients, as reported)	97–98	day 8 / day 13	day 22	day 12
Culture-positive joints only	39	day 9 / day 16	day 35	day 15
Recognized pathogen only	28	day 10 / day 16	day 27	day 15
Exclude the nine crystal-positive joints	88–89	day 7 / day 12	day 23	day 12
Exclude the six patients with documented arthroscopic surgery elsewhere for the	91–92	day 8 / day 13	day 23	day 13

Analysis	Patients (n)	CRP <5 / <3 mg/dL	NLR <3	CAR <1.0
presenting episode				
Exclude all seven patients with documented surgery elsewhere for the presenting episode	90–91	day 8 / day 14	day 23	day 13

Panel D. Repeat operation following clinical signs of persistent or recurrent infection (exploratory; too few events for inference).

Marker	Patients with clinical-sign repeat operation (n)	Interaction β per 10 days	*p*
CRP	7	0.012	0.95
NLR	7	−0.082	0.42
CAR	7	0.113	0.66

Group-by-time interaction (linear day, random slope), censored at the first repeat operation: patients whose first repeat followed clinical signs (effusion, wound drainage, fever, or pain) versus all others. Reported for completeness only.

Milestone day = first day the fitted geometric-mean curve crossed the reference; CRP <1 mg/dL, NLR <2, and CAR <0.5 (not reached in the primary analysis) are omitted. Across Panels A and B, primary milestones moved by at most three days (CRP <5 mg/dL unchanged at day 8). Panel C addresses diagnostic certainty (59 of 98 joints culture-negative) and prior surgery elsewhere: excluding the nine crystal-positive joints or the patients operated on elsewhere moved milestones by at most one day; culture-confirmed joints crossed later (CRP by one to three days, CAR three, NLR five to 13), so cohort composition influences the estimated tempo and the whole-cohort curve is not a reference for microbiologically confirmed infection. These subgroups (39 and 28 patients) are a robustness check, not subgroup reference curves. All Panel C models used 4 degrees of freedom on the full-rank basis (submitted version: culture-positive CRP fitted at 5, CRP <3 mg/dL day 17). CAR, CRP-to-albumin ratio; CRP, C-reactive protein; I&D, irrigation and debridement; NLR, neutrophil-to-lymphocyte ratio.

Supplementary Table S4. Fitted geometric-mean marker values at landmark postoperative days, with 95% confidence and prediction intervals

Postoperative day	CRP (mg/dL), fitted (95% CI) [95% PI]	NLR, fitted (95% CI) [95% PI]	CAR, fitted (95% CI) [95% PI]
3	8.4 (7.3–9.6) [1.1–67.2]	6.2 (5.6–6.9) [1.6–23.3]	2.75 (2.37–3.18) [0.31–24.45]
5	6.6 (5.7–7.6) [0.8–52.7]	5.6 (5.0–6.3) [1.5–21.0]	2.13 (1.80–2.48) [0.24–18.91]
7	5.2 (4.4–6.1) [0.7–41.9]	5.0 (4.4–5.7) [1.3–19.0]	1.67 (1.38–1.98) [0.19–14.84]
14	2.8 (2.1–3.4) [0.3–22.2]	3.7 (3.2–4.3) [1.0–14.0]	0.85 (0.65–1.09) [0.10–7.64]
21	2.1 (1.5–2.7) [0.3–16.6]	3.1 (2.6–3.5) [0.8–11.5]	0.64 (0.47–0.85) [0.07–5.74]
28	1.9 (1.4–2.5) [0.2–15.2]	2.8 (2.4–3.2) [0.7–10.5]	0.59 (0.42–0.81) [0.07–5.39]
42	1.6 (0.9–2.5) [0.2–13.7]	2.8 (2.2–3.4) [0.7–10.6]	0.55 (0.31–0.85) [0.06–5.12]

Primary random-intercept model (Fig. 2): fitted geometric mean (95% CI, cluster bootstrap) [95% PI for an individual patient], as effect-size anchors for landmark studies. In later windows the fitted means lie below the observed window medians (Table 2 Panel C). Milestone intervals from the confidence band: CRP <5 mg/dL day 8 (6–10), <3 mg/dL day 13 (11–17), CAR <1.0 day 12 (10–16), NLR <3 day 22 with the band compatible with 3 from day 16; the NLR upper limit never falls below 3 (minimum 3.1 at day 34), so the NLR milestone is a crossing of the fitted curve, not of the band. CRP in mg/dL (1 mg/dL = 10 mg/L); CAR with CRP in mg/dL. CAR, CRP-to-albumin ratio; CI, confidence interval; CRP, C-reactive protein; NLR, neutrophil-to-lymphocyte ratio; PI, prediction interval.

Supplementary Table S5. Synovial fluid and preoperative serum markers by microbiological status

Measure	Culture-positive (n = 39)	Culture-negative (n = 59)	*p*	Recognized pathogen (n = 28)	Other (n = 70)	*p*
Synovial white-cell count (cells/µL)	57,586 [31,443–111,478] (34)	52,850 [35,755–81,712] (56)	0.72	62,541 [35,397–139,208] (24)	52,835 [35,476–77,213] (66)	0.26
Synovial white-cell count >50,000/µL, n/N	20/34	31/56	0.83	15/24	36/66	0.63
Synovial neutrophils (%)	92 [88–95] (35)	93 [87–95] (56)	0.83	92 [88–95] (25)	93 [87–96] (66)	0.37
Preoperative CRP (mg/dL)	15.3 [7.8–23.1] (38)	9.8 [5.4–16.9] (58)	0.023	17.1 [9.3–23.5] (27)	10.2 [5.2–16.9] (69)	0.014
Preoperative NLR	10.9 [6.8–14.5] (39)	5.9 [4.0–9.1] (59)	<0.001	11.4 [7.3–16.5] (28)	6.3 [4.4–9.3] (70)	<0.001
Preoperative CAR	5.2 [2.0–7.5] (37)	2.8 [1.6–4.7] (57)	0.014	6.0 [2.5–8.5] (26)	3.0 [1.5–4.9] (68)	0.004
Age (years)	77.0 [66.0–84.5]	78.0 [66.0–84.0]	0.89	75.0 [66.0–82.2]	80.0 [66.0–84.8]	0.53
Diabetes mellitus, n/N	16/39	19/59	0.40	12/28	23/70	0.36

Median [IQR] (n) or n/N; Mann–Whitney U and Fisher exact tests, unadjusted and descriptive. Crystal-positive joints: three of nine culture-positive (two recognized pathogens); synovial white-cell count median 68,700 cells/µL [66,770–90,200]. Culture categories as in Table 1. CAR, CRP-to-albumin ratio; CRP, C-reactive protein; NLR, neutrophil-to-lymphocyte ratio.

Supplementary Table S6. Reoperations evaluated for repeat-I&D classification

ID	Postoperative day	Recorded procedure	Timing	Documented indication	Counted as repeat I&D
R1	4	Debridement and VAC change	Index admission	Clinical signs	Yes
R2	7	Open I&D	Index admission	CRP rise only	Yes
R3	8	Open I&D	Index admission	CRP rise only	Yes
R3	22	I&D	Index admission	CRP rise only	Yes (second repeat)

ID	Postoperative day	Recorded procedure	Timing	Documented indication	Counted as repeat I&D
R4	12	Revision I&D	Index admission	CRP rise only	Yes
R5	14	I&D	Index admission	CRP rise only	Yes
R6	17	I&D	Index admission	Clinical signs	Yes
R7	21	I&D	Index admission	Clinical signs	Yes
R8	22	Open I&D	Index admission	CRP rise only	Yes
R9	41	Open I&D	After readmission	Clinical signs	Yes
R10	48	Open I&D	After readmission	Clinical signs	Yes
R11	49	I&D with VAC	After readmission	Clinical signs	Yes
R12	65	Open I&D	After readmission	Clinical signs	Yes
M1	41	Brisement (manipulation under anesthesia)	After readmission (2 days)	Stiffness	No
M2	56	Manipulation under anesthesia	After readmission (2 days)	Stiffness	No

Anonymous sequential identifiers (R, repeat I&D, ordered by first repeat; M, manipulation). Salvage procedures (one amputation and two arthroplasties, 15 to 46 months after the index operation) are listed in Table 3. Procedure as recorded in the institutional operative schedule; timing and indication from the admission records and the operative and progress notes. CRP rise only = renewed CRP rise after its initial decline without other documented signs; clinical signs = effusion, wound drainage, fever, or pain. The submitted manuscript had counted M1 and M2 as repeat I&D (14 patients); both were closed manipulations for stiffness (manipulation under anesthesia and brisement), without arthrotomy or debridement according to the operative and instrument records, during two-day readmissions, and are not counted (12 patients). CRP, C-reactive protein; I&D, irrigation and debridement; VAC, vacuum-assisted closure.

Supplementary Figure S1. Random-intercept versus random-slope trajectories

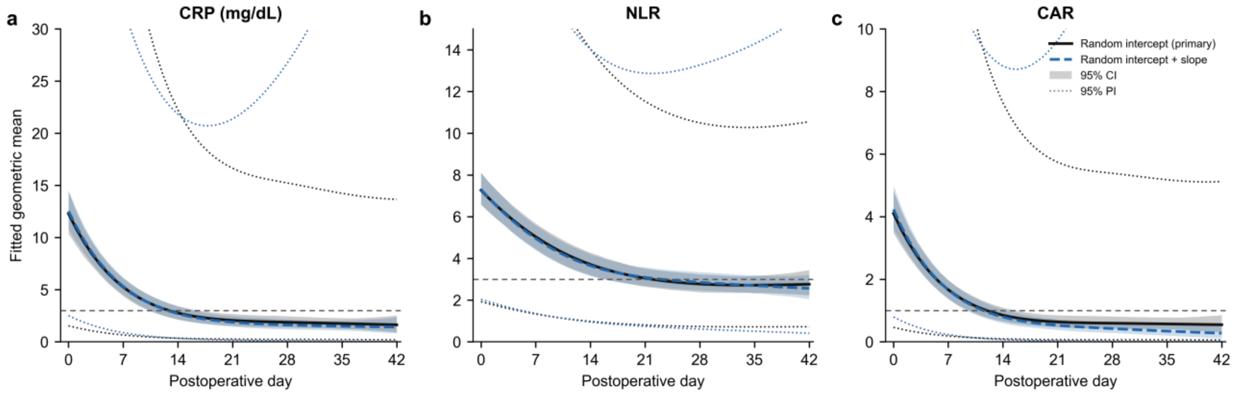

Fitted geometric mean (lines), 95% confidence interval (shaded; cluster bootstrap), and 95% prediction interval (dotted) for (a) CRP, (b) NLR, and (c) CAR: random intercept (black, solid) versus random intercept plus slope (blue, dashed). Dashed horizontal line, reference value (CRP 3 mg/dL; NLR 3; CAR 1.0); upper prediction bounds truncated. CAR, CRP-to-albumin ratio; CRP, C-reactive protein; NLR, neutrophil-to-lymphocyte ratio.

Supplementary Figure S2. Effect of censoring at the first repeat operation

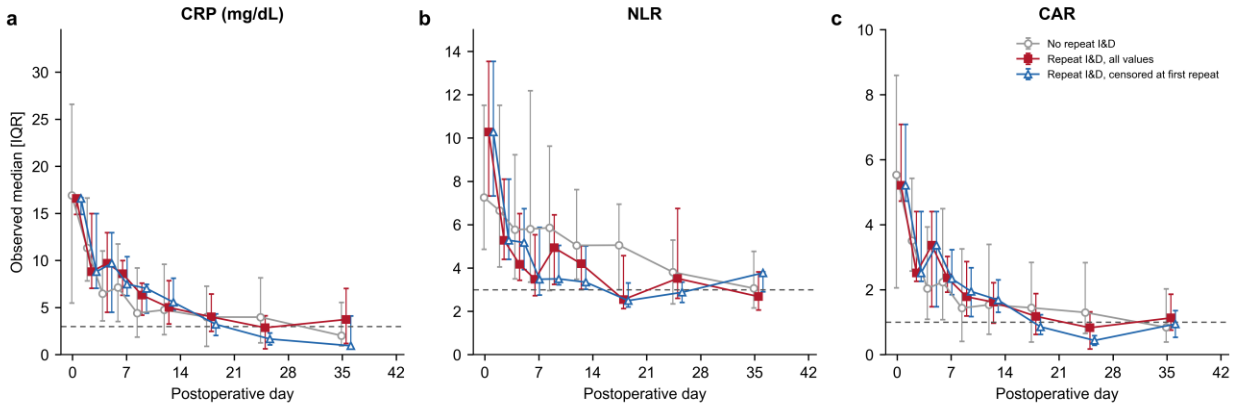

Observed median [IQR] by window for (a) CRP, (b) NLR, and (c) CAR: 12 repeat-I&D patients with all measurements (red squares) and with measurements before the first repeat operation only (blue triangles), versus 86 patients without repeat I&D (grey circles). Post-reoperation values hold up the uncensored medians; after censoring, few repeat patients contribute beyond day 21 (2–3 in the last two CRP windows), so late censored medians are imprecise. Model-free by design. CAR, CRP-to-albumin ratio; CRP, C-reactive protein; I&D, irrigation and debridement; NLR, neutrophil-to-lymphocyte ratio.
